# An exploratory cluster randomised controlled trial of knowledge translation strategies to support evidence-informed decision-making in local governments (The KT4LG study)

**DOI:** 10.1186/1471-2458-11-34

**Published:** 2011-01-13

**Authors:** Elizabeth Waters, Rebecca Armstrong, Boyd Swinburn, Laurence Moore, Maureen Dobbins, Laurie Anderson, Mark Petticrew, Rachel Clark, Rebecca Conning, Marj Moodie, Robert Carter

**Affiliations:** 1 Jack Brockhoff Child Health and Wellbeing Program, McCaughey Centre: Melbourne School of Population Health, University of Melbourne, Level 5/207 Bouverie Street, Carlton, Victoria, Australia; 2 Population Health Strategic Research, Deakin University, 221 Burwood Highway, Burwood, Victoria, Australia; 3 Cardiff Institute of Society and Health, School of Social Sciences, Cardiff University, 1-3 Museum Place, Cardiff, UK; 4 School of Nursing, McMaster University, Main Street West, Hamilton, Ontario, Canada; 5 Washington State Institute for Public Policy, Olympia, Washington, USA; 6 London School of Hygiene and Tropical Medicine, University of London, Keppel St, London, UK; 7 Deakin Health Economics, School of Health and Social Development, Deakin University, Burwood Highway, Burwood, Victoria, Australia

## Abstract

**Background:**

Childhood overweight and obesity is the most prevalent and, arguably, politically complex child health problem internationally. Governments, communities and industry have important roles to play, and are increasingly expected to deliver an evidence-informed system-wide prevention program. However, efforts are impeded by a lack of organisational access to and use of research evidence. This study aims to identify feasible, acceptable and ideally, effective knowledge translation (KT) strategies to increase evidence-informed decision-making in local governments, within the context of childhood obesity prevention as a national policy priority.

**Methods/Design:**

This paper describes the methods for KT4LG, a cluster randomised controlled trial which is exploratory in nature, given the limited evidence base and methodological advances. KT4LG aims to examine a program of KT strategies to increase the use of research evidence in informing public health decisions in local governments. KT4LG will also assess the feasibility and acceptability of the intervention. The intervention program comprises a facilitated program of evidence awareness, access to tailored research evidence, critical appraisal skills development, networking and evidence summaries and will be compared to provision of evidence summaries alone in the control program. 28 local governments were randomised to intervention or control, using computer generated numbers, stratified by budget tertile (high, medium or low). Questionnaires will be used to measure impact, costs, and outcomes, and key informant interviews will be used to examine processes, feasibility, and experiences. Policy tracer studies will be included to examine impact of intervention on policies within relevant government policy documents.

**Discussion:**

Knowledge translation intervention studies with a focus on public health and prevention are very few in number. Thus, this study will provide essential data on the experience of program implementation and evaluation of a system-integrated intervention program employed within the local government public health context. Standardised programs of system, organisational and individual KT strategies have not been described or rigorously evaluated. As such, the findings will make a significant contribution to understanding whether a facilitated program of KT strategies hold promise for facilitating evidence-informed public health decision making within complex multisectoral government organisations.

**Trial registration:**

Australia and New Zealand Clinical Trials Register (ANZCTR): ACTRN12609000953235

## Background

Obesity is a significant global public health issue. In Australia, a recent national health survey found 25% of children aged 5-17 years have been classified as overweight or obese. Childhood obesity has been linked to a range of social and physical health problems in later life including type 2 diabetes [1]. As a result, data suggests that the financial cost of obesity in 2008 was $8.283 Billion AUD [2]. Such forecasts are fuelling political imperatives to implement obesity prevention strategies, particularly for primary-school aged children. Developing strategies that incorporate new knowledge into policy development is essential.

Local governments in Australia have responsibility for a range of local infrastructure and activities including maternal and child health programs, child care, kindergartens, recreation facilities, parks, planning and building, traffic management and food regulations. Due to this diverse portfolio, local government has the potential to make a significant contribution to obesity prevention in Australia [3,4]. To be most effective however, strategies undertaken by local government need to be informed by the best available research evidence. Research evidence can be broadly defined as including descriptive evidence of prevalence and risk, evidence of intervention or program effectiveness and evidence about implementation of these interventions (for whom interventions work or not, in what circumstances and why) [5-7]. The crucial role of evidence has been formally acknowledged in this arena in Victoria with the Victorian Health and Wellbeing Act 2008, stipulating that local governments use evidence to inform their Municipal Public Health Plans [8]. The use of research evidence is important not only in terms of identifying the combinations of strategies that impact on obesity, but also how well they work, for which sub-groups, the potential for harm, the mechanisms needed to support strategies, and associated cost [9]. The use of research evidence to inform decision-making, and the development of strategies to support this process is therefore crucial.

Evidence-informed decision-making (EIDM) involves integrating the best available research evidence with contextual factors including community preferences, local health issues, political preferences and public health resources[10]. The benefits of EIDM include:

▪ adoption of effective and cost-efficient interventions,

▪ prudent use of scarce resources,

▪ improved client satisfaction, and

▪ better health outcomes for individuals and communities [10].

In order for evidence-informed decision-making to occur efficiently and effectively, a series of mechanisms are required. Researchers and decision-makers need to work in partnership to fund and conduct research that addresses key policy questions; research needs to be conceptualised, conducted and communicated in a way that is meaningful to decision-makers[11]; and research evidence needs to be accessed, assessed and appropriately [12] applied by decision-makers within a complex political system. (Appropriate use of research evidence to inform decision-making refers to an unbiased assessment of the evidence-base rather than using research evidence to support a position or decision that been made in the absence of evidence)

While decision-makers are under increasing pressure to use research evidence to inform their decisions, significant barriers have been identified. These include absence of personal contact between researchers and policy-makers and practitioners, lack of time and resources, organisational structures and processes, timeliness of research, poor quality or limited availability of research, and political influence [11-14]. To address these barriers, a range of strategies, often conceptualised as knowledge translation (KT) have been described and in some cases implemented. KT strategies range from researcher focused interventions (often designed to support the dissemination of research findings), decision-maker focused interventions (often designed to change practices and behaviours related to the integration of research evidence into decision-making processes) and interventions designed to create partnerships between researchers and decision-makers (where questions of mutual interest are identified, research is conducted in partnership and the research is used to inform policy-level decisions) [15]. These have also been categorised as interventions that encourage push, pull and exchange of evidence [16]. This study focuses on the application of decision-maker focused interventions, that is, those that facilitate decision-makers to access and use research evidence to inform their decisions.

Despite the fervour around KT in public health, very few rigorous studies have been or are being conducted [17]. As a result, it is likely that strategies have been either modified from those conducted in clinical environments or informed by the plethora of studies (of varying quality) that outline barriers and facilitators to KT in public health environments [16]. Much of the work to date has occurred in Canada where the government has actively invested in exploring and facilitating KT [18]. This exploratory trial will assess the potential effectiveness, as well as feasibility and acceptability, of a multi-component KT intervention in increasing the use of research evidence to support decision making in local governments. The trial is informed by a series of preliminary projects including a state-wide survey, a systematic review and key informant interviews, which will be published elsewhere. This paper describes the methods of the trial in detail.

## Aims and objectives

**Figure 1:**
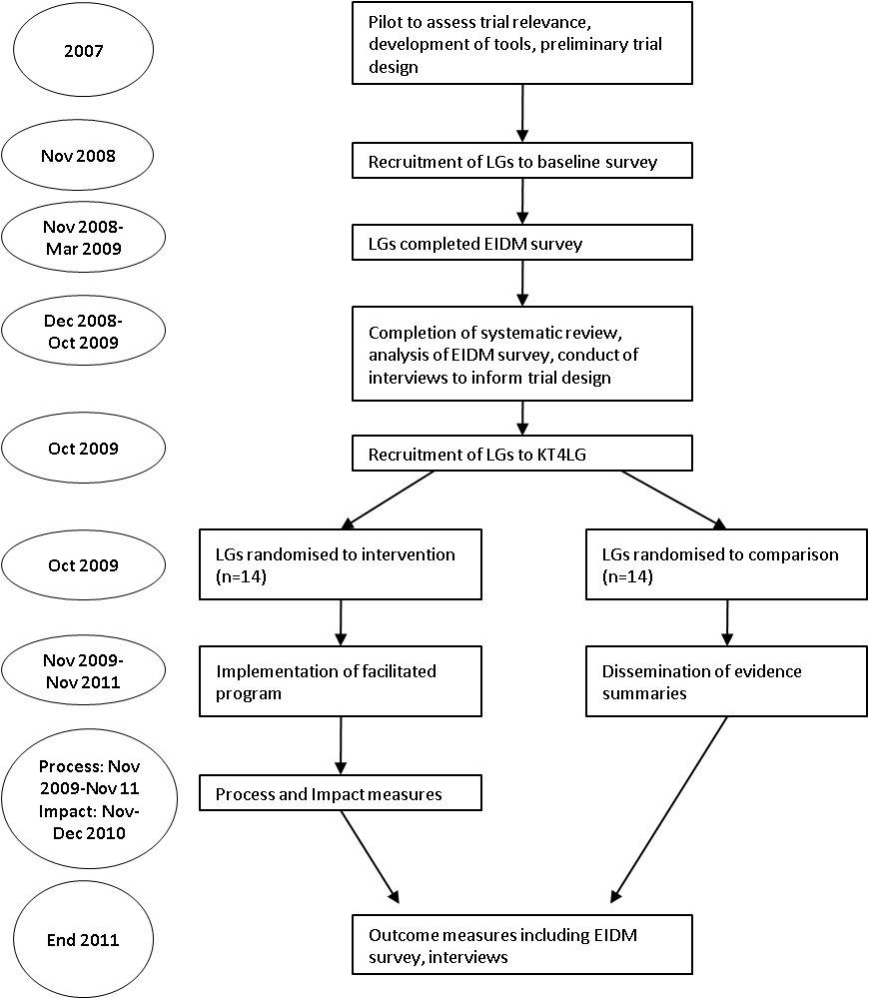
**Proposed study timetable**.

1. What KT strategies show promise to increase the use of research evidence in policy and program decision-making in local governments?

2. How can we measure evidence use and the key sub-components of evidence use (as outlined in Figure 1) that are to be targeted by the intervention, at decision-maker and council level?

3. What are estimates of intra-cluster correlation by council of outcomes and of the change in such outcomes that a larger trial would need to be powered to detect?

In order to answer these questions this research program has 2 key aims:

1. Undertake an exploratory cluster randomised controlled trial to identify acceptability of the multi-component knowledge translation and exchange model, identify potential modifications, estimate key parameters required for a full scale trial including recruitment procedures, recruitment and retention rates, variation in outcome measures, intra-cluster correlation coefficients, and estimate feasible effect sizes and intervention costs for a full scale trial.

2. Develop recommendations about KT models applicable to local government settings and specific recommendations for the value and design of a large scale trial.

## Methods/Design

### Study design

The study design is a cluster randomised controlled trial where local governments (councils) are randomised to intervention or control.

The intervention consists of two core components: 1. improved access to research evidence via provision of summaries of intervention research on topics of relevance in childhood obesity prevention for local government; and 2. building capacity of local governments to use research evidence to inform public health decision-making by providing facilitated support. Control (comparison) councils will receive component 1 and intervention councils will receive both components. The duration of the intervention will be two years. Figure 1 outlines the proposed study timetable.

#### Intervention councils

Facilitated support will be managed by a program coordinator and consist of:

▪ Increased access to research evidence through supplementing evidence summaries with support to assess applicability of evidence to local context,

▪ Professional development sessions with a focus on acquiring, assessing and applying research evidence to local context,

▪ Explore and implement strategies aimed at the development of an organisational culture that supports evidence-informed decision-making within local governments.

#### Comparison councils

The comparison councils will receive access to summaries of intervention research. These will be circulated by email to all participating councils.

This research program will incorporate two of the phases in the UK Medical Research Council's (MRC) framework for evaluating complex interventions: Phase I and Phase II [19]. The developmental research program involved development of the intervention's components, based on existing evidence, survey data and key informant interviews.

This paper reports on the design of the intervention trial, which is being conducted in 28 councils (14 randomised to participate in the intervention and 14 to act as comparison councils). This will inform the development of a case for a possible future Phase III trial [19].

Ethical approval for this project was granted by the University of Melbourne Human Ethics Committee [ref 0722362).

### Recruitment and randomisation of local governments

#### Baseline survey

All 79 Victorian local governments were invited to participate in a state-wide baseline survey. Individuals that were employed by Victorian local governments and involved in public health were eligible for participation. Up to four people from each local government were invited to complete an online survey. The survey aimed to elicit how local governments currently make decisions, the contribution of research evidence to decision-making, the facilitators and barriers to EIDM to identify strategies already in place that support evidence-informed decision-making and to explore additional strategies that might support EIDM. One hundred and thirty six responses from 45 local governments were received. Reasons for non-participation included workload, staffing issues (e.g. positions currently vacant), and a recent bushfire disaster. Many of those unable to participate said that they supported the study and asked to be kept informed of the outcomes. Only those local governments who agreed to participate in the initial survey were invited to participate in the trial.

#### Intervention study

All 45 councils who completed the pre-trial baseline survey were then invited to participate in the trial. Contact was made initially by phone and then a memorandum of understanding (MOU) was emailed for the key contact to complete. The MOU articulated the roles and responsibilities of both councils and researchers. Members of the research team were available to answer queries, either by telephone or site visit during the recruitment stage. Follow-up telephone calls were made to unresponsive key contacts. Councils who declined participation were removed from the sample and not contacted again for recruitment purposes.

#### Randomisation

Stratified block randomisation was used to randomly allocate participating councils to intervention or comparison group. Strata were defined by tertile of council's annual budget. Analysis of the baseline survey revealed a linear relationship between budget and population size which suggested that either could be selected as the stratification variables. Given the anticipated resource implications of practicing EIDM (e.g. staffing resources, access to internet and other resources), council budget was deemed to be most appropriate. Each intervention council then nominated a number of staff to participate in the intervention, ideally those who participate in public health planning. Once participation was agreed and a memorandum of understanding was signed, the programme coordinator emailed the trial manager with the name and annual budget of the council. The trial manager then emailed the trial statistician with the stratum (but not name) of the council. The statistician then allocated the council (blind to its identity) to intervention or comparison on the basis of a randomisation sequence of block size 4 generated and held by the trial statistician and concealed to all other parties. The trial manager logged the group allocation on the trial database and informed the program coordinator.

### Sample size

Given that the outcome measures had not previously been employed precise sample size calculations, whilst broadly estimated a priori, were unable to be undertaken in the absence of estimates of standard deviation and intra-cluster correlation. However, this study will provide estimates of SD and ICC.

### Outcome measures

#### Primary outcome measures

1. Research evidence use by LGs in intervention and comparison councils

#### Secondary outcome measures

1. Access to research evidence

2. Confidence using research evidence

3. Organisational culture for evidence-informed decision-making

4. Influence of research evidence on public health decisions

5. Usefulness of research evidence in making public health decisions

### Measurement of outcomes

Outcome evaluation will include the EIDM survey and a series of policy tracer studies. Outcome evaluation will focus on research evidence generally and research evidence specific to obesity prevention.

### EIDM survey and key informant interviews

Following the intervention period, all participants will be invited to complete an on-line survey. This will include those who have actively participated in the intervention components as well as representatives nominated from comparison councils. Unresponsive participants will be contacted once with an email reminder. A purposive sample of participants in both comparison and intervention groups will also be invited to participate in key informant interviews.

The post intervention survey will comprise measures developed in the analysis of the baseline survey as outcome measures, testing the sensitivity of the measures and aiming to obtain estimates of standard deviation and intra class correlation. Outcome measures will include access, confidence, and organisational culture in addition to the perceived influence and usefulness of a range of sources of evidence. Given the complexities associated with measuring research use, a triangulated approach to measuring a range of constructs of research use will be used.

The background and development of the EIDM survey and results of the baseline survey will be published separately, however in summary it was based on two measurement tools [20,21], the first previously assessed for reliability and validity amongst public health decision-makers in Canada [22], and the second developed for use with public health decision-makers working in local authorities in the UK [20]. These measures were modified to be relevant for use in local government in Victoria, based on related literature [22-25]. The EIDM questionnaire was pilot tested with five individuals who had either worked with or in local government.

### Policy tracer studies

Policy tracer studies will be conducted post intervention to examine policy change and track the influence of research retrospectively. These studies will also examine the feasibility and utility of using such methods to provide outcome data, and if successful to investigate the influence of research on policy [26]. The tracer studies will examine policy change and track the influence of research retrospectively. They will be conducted in 4 purposively selected intervention councils at the conclusion of the intervention. The sampling frame will consider council size and structure and engagement with KT4LG as monitored by the study team. Councils who agree to participate will select a policy change and in-depth interviews with key actors across council will be conducted by the KB. The focus of the interview will be to explore the lead-up to the decision, why the decision was made, who was involved, and what evidence was useful and influential. This will be combined with document analysis of meeting minutes, policy drafts and project plans where available.

### Impact evaluation

Regular monthly meetings and training sessions with intervention councils will be used as opportunities to explore the impact of the value of the intervention. Intermediary outcomes of interest include skills in using research evidence and combining with other sources of evidence, access to research evidence, and confidence in using research evidence. Site visits will be conducted at the one-year point of the intervention with participating councils (November 2010) and repeated again at the conclusion of the intervention (November 2011). During these visits, tools that focus on exploring stages and levels of evidence utilisation at both individual and organisational levels will be used to assess research use [27,28]. Data will be collected by the KB during these face to face meetings and analysed by RA under the guidance of the research team. Evaluation of training sessions will also be conducted and will contribute to the impact evaluation.

### Process evaluation/measures

The process evaluation will seek to capture information related to the intensity (dose), fidelity and reach of the intervention. Measures of context, staff turnover, barriers, facilitators, quality of engagement, which are likely to be useful in assessing acceptability and helping to modify the logic model and intervention content/delivery will be captured. An Access database has been developed to collect this data.

Measures of adoption will include:

▪ Number of councils involved - intervention and control

▪ Number of councils attending each training session

▪ Number of councils adopting our materials, frameworks (including EIDM and also determinants approach to obesity prevention)

▪ Other organizations adopting intervention materials

▪ Departments across council adopting our materials, frameworks

▪ Number of councils participating in monthly contact

▪ Number of councils that don't participate and why

▪ Demographics of participating councils

▪ Cost of the intervention to councils

Measures of reach will include:

▪ Number of participants from each council attending training sessions

▪ Number of people participating in monthly contact (which department)

▪ Number of people attending research symposium

▪ Number of additional stakeholders involved

▪ Resources distributed

▪ Characteristics of participants (job description, position title)

▪ What helped and hindered participation by individuals

▪ Characteristics of non-participants

Measures of implementation will include:

▪ Number of monthly contacts

▪ Number of training sessions

▪ Number of forums

▪ Requests for attendance at meetings

▪ Participant satisfaction (evaluation of training sessions and research symposium)

▪ Delivery of training sessions compared to expected delivery

▪ Delivery of monthly contact compared to expected delivery

▪ Why/why not are resources used by participants

▪ Cost of implementation (to researchers)

▪ Timeliness of intervention delivery (do we stick to our timeline)

▪ Quality of delivery

▪ Any issues raised during implementation - and how were these addressed

▪ Delivery of intervention components as planned

### Economic evaluation

In addition, an economic evaluation will help to answer the key questions of whether the intervention is affordable (i.e. gross/net cost) and whether it offers value-for money. Pathway analysis will be used to identify the resources associated with each arm. These resources will be valued using the most up-to-date sources for the 2010 reference year. The incremental or net intervention cost will equal the difference in costs between the two intervention arms. The consequences of the intervention will be measured in terms of willingness-to-pay (WTP) in a cost-benefit analysis (CBA). A CBA compares the net costs of the intervention to the dollar value of the identified outcomes.

### Data management and analysis

Quantitative data will be collected using the EIDM survey. Participant responses will be collected using Survey Monkey [29] and then transferred to Stata for analysis [30].Baseline characteristics of intervention and comparison councils and staff will be compared. Summary statistics for each outcome, broken down by group, will be presented at follow-up, with 95% confidence intervals appropriately adjusted for clustering (Stata svy procedures).

The primary analysis for assessment of the effect of KT4LG on primary outcomes would be ascertained in a large scale trial using a council-level weighted regression model with the stratification variable (budget tertile) and baseline measures included as covariates, It is acknowledged that the sample size in this exploratory trial is small and therefore provide imprecise effect estimates (and 95% confidence intervals). More usefully, the data from this study will be analysed to provide estimates of the intracluster correlation for each outcome, which will be calculated using analysis of variance (Stata loneway), and of standard deviations and correlations of baseline and follow up measures (council and staff level).

Qualitative data will be collected in a variety of ways but will predominantly involve key informant interviews and process diaries maintained by the KB. Key informant interviews will be tape-recorded and transcribed verbatim. Given the lack of one theoretical approach to guide this work, a grounded theory approach will be used to inform qualitative data analysis [31-33]. The qualitative data will be coded and categorised using N-Vivo software [34] to identify emerging patterns and themes. While this study seeks to influence the use of research evidence it is acknowledged that other types of knowledge also inform decision-making. The process evaluation will collect this information and will also explore the influences on decision-makers [35].

Data collection for the economic evaluation will be closely integrated in the process evaluation, described earlier. A questionnaire will be administered to stakeholders from each of the participating councils (both intervention arms) at the conclusion of the intervention. It will ask a series of questions to ascertain the maximum amount which a council would be willing to pay (WTP) to have access to the KT4LG intervention. The questionnaire will be piloted in face-to-face format as part of the tracer study interviews. The usefulness of the WTP method in assessing value attached to the KT4LG intervention will be assessed both for specific examples (the tracer studies) and as a generic question. To avoid operator bias, the delivery of the intervention and the collection and analysis of data will be separated. Where data is collected, the KB will be responsible for data collection but will not be involved in data analysis. The project team will be supported by the scientific research team comprised of the chief and associate investigators who will advise on scientific and ethical issues. To monitor adverse effects of the intervention, we will be examining this issue with councils at mid point and completion of the study.

## Discussion

This study will make a significant contribution to better understanding how to support evidence-informed policy and practice decisions in local public health environments. This is important given the increasing pressure for policy makers to use evidence to inform their decision-making processes. As one of few rigorous evaluations in this area, it will build on a limited body of knowledge relevant to public health.

The study uses a rigorous design to explore an area that is complex and not well understood. There is likely to be considerable heterogeneity between local governments. In addition, there is likely to be staff turnover within councils as the intervention is implemented. These issues have been addressed in the study design.

Identification of outcome measures to assess the effectiveness of KT will be informed by the literature and from findings from Dobbin's Canadian study [22]. Further research on relevant outcome measures is needed and the research team intends to secure additional funding in this area.

This intervention, designed to support evidence-informed decision-making, is timely and relevant to the current context in which policy decisions are being made. The intervention will contribute to better understanding how evidence can be is used to informed public health decisions, what the barriers are, and identify at what level interventions are needed to support transparent and well informed decisions.

## List of abbreviations

CEOs: Chief Executive Officers; CRCT: cluster randomised controlled trial; EIDM: evidence-informed decision-making; KB: knowledge broker; KT: knowledge translation; MAV: Municipal Association of Victoria; VicHealth: Victorian Health Promotion Foundation; MOU: Memorandum of understanding.

## Competing interests

The authors declare that they have no competing interests.

## Authors' contributions

All authors were actively involved in the development and design of the study. EW, and RA drafted the manuscript. EW is the principal Chief Investigator and leads the study, RA is the project manager. LM, MD, LA, RC, BS all contributed to the draft of this paper. All authors signed off on the final version.

## Pre-publication history

The pre-publication history for this paper can be accessed here:

http://www.biomedcentral.com/1471-2458/11/34/prepub
